# Intermediate conformations of CD4-bound HIV-1 Env heterotrimers

**DOI:** 10.1038/s41586-023-06639-8

**Published:** 2023-11-22

**Authors:** Kim-Marie A. Dam, Chengcheng Fan, Zhi Yang, Pamela J. Bjorkman

**Affiliations:** 1 Division of Biology and Biological Engineering, California Institute of Technology, Pasadena, CA USA; 2 Present Address: Department of Molecular and Cell Biology, University of California, Berkeley, CA USA

**Keywords:** Cryoelectron microscopy, Virus structures

## Abstract

HIV-1 envelope (Env) exhibits distinct conformational changes in response to host receptor (CD4) engagement. Env, a trimer of gp120 and gp41 heterodimers, has been structurally characterized in a closed, prefusion conformation with closely associated gp120s and coreceptor binding sites on gp120 V3 hidden by V1V2 loops^1–4^ and in fully saturated CD4-bound open Env conformations with changes including outwardly rotated gp120s and displaced V1V2 loops^3–9^. To investigate changes resulting from substoichiometric CD4 binding, we solved single-particle cryo-electron microscopy (cryo-EM) structures of soluble, native-like heterotrimeric Envs bound to one or two CD4 molecules. Most of the Env trimers bound to one CD4 adopted the closed, prefusion Env state, with a minority exhibiting a heterogeneous partially open Env conformation. When bound to two CD4s, the CD4-bound gp120s exhibited an open Env conformation including a four-stranded gp120 bridging sheet and displaced gp120 V1V2 loops that expose the coreceptor sites on V3. The third gp120 adopted an intermediate, occluded-open state^10^ that showed gp120 outward rotation but maintained the prefusion three-stranded gp120 bridging sheet with only partial V1V2 displacement and V3 exposure. We conclude that most of the engagements with one CD4 molecule were insufficient to stimulate CD4-induced conformational changes, whereas binding two CD4 molecules led to Env opening in CD4-bound protomers only. The substoichiometric CD4-bound soluble Env heterotrimer structures resembled counterparts derived from a cryo-electron tomography study of complexes between virion-bound Envs and membrane-anchored CD4 (ref. ^11^), validating their physiological relevance. Together, these results illuminate intermediate conformations of HIV-1 Env and illustrate its structural plasticity.

## Abstract (web summary)

Single-particle cryo-electron microscopy structures of HIV-1 envelope (Env) trimers bound to one or two CD4 receptors identify intermediate Env conformations that precede host–virus fusion and inform the design of therapeutics to prevent HIV-1 infection.

## Main

The HIV-1 Env glycoprotein, a heavily glycosylated homotrimer containing gp120 and gp41 subunits, mediates entry into host cells to initiate infection^12^. On the surface of virions, Env adopts a closed, prefusion conformation similar to that observed in soluble native-like Env trimer ectodomains^1–4,13^. The viral entry process is initiated when gp120s bind to the host receptor, CD4, at the CD4-binding site (CD4bs) located distal to the Env apex on the sides of each of the three gp120s^5–9^. This triggers conformational changes in gp120 that expose the gp120 V3 coreceptor binding site, which is occluded in the prefusion conformation beneath gp120 V1V2 loops^5–9^. Coreceptor binding results in further conformational changes that lead to insertion of the gp41 fusion peptide into the host cell membrane and fusion of viral and host membranes^1,10^.

X-ray crystallography and single-particle cryo-EM structures have enabled characterization of soluble versions of HIV-1 Envs^14^ in closed, prefusion^1,2^, CD4-bound open^5–7^, and intermediate partially open conformations^5,9,10^. Several studies have demonstrated that the native-like soluble Envs (SOSIPs)^14^ used for structural studies resemble virion-bound Envs, indicating that these conformations may be relevant to the viral Env entry process^3,4,14–17^. The closed, prefusion Env conformation is characterized by gp120 V1V2 loops interacting around the trimer apex, thereby shielding the coreceptor binding sites on the V3 loops^1,2,18^. CD4-bound open Env trimer structures revealed receptor-induced changes in which the gp120 subunits rotated outwards, the V1V2 loops were displaced from the apex by approximately 40 Å to the sides of Env, and the coreceptor binding site on each V3 was exposed and became mostly disordered^5–9^ (Supplementary Video 1). This process also converted the closed, prefusion conformation three-stranded gp120 bridging sheet composed of the β20, β21 and β3 β-strands^1^ to a four-stranded antiparallel β-sheet in which strand β2, whose residues are located in a proximal helix in the closed, prefusion formation, is intercalated between strands β21 and β3 (refs. ^1,5,6,9^). Intermediate Env conformations include occluded-open^5,10^ and partially open conformations^9,19^. In the occluded-open conformation observed in trimer complexes with the CD4bs antibody b12 (ref. ^5^) and similar antibodies raised in vaccinated non-human primates^10^, the gp120 subunits were outwardly rotated from the central trimer axis as in CD4-bound open conformations, but V1V2 displacement and V3 exposure did not occur, and the prefusion three-stranded gp120 β-sheet was maintained^5,10^. In partially open Env conformations, CD4 binding led to the characteristic CD4-induced structural changes in gp120, but subsequent binding of the gp120–gp41 interface antibody 8ANC195 led to partial closure of the gp120s^9^.

A prevailing enigma about Env conformational changes and the role of CD4 in initiating the fusion process concerns whether the gp120/gp41 protomers that form the Env trimer behave cooperatively or independently during receptor-induced transformations. This information would reveal how many CD4 receptor and CCR5 coreceptor molecules are needed to engage each Env trimer to initiate fusion and further explain Env function as it relates to virus infectivity, thereby informing the design of entry inhibitors and mechanisms of antibody neutralization and fusion. To investigate the role of receptor stoichiometry in CD4-induced conformational changes in HIV-1 Env, we designed soluble Env heterotrimers that can bind only one or only two CD4 receptors for comparisons with Env homotrimers binding either zero CD4s (closed, prefusion trimers) or three CD4s (fully saturated CD4-bound open trimers). Using single-particle cryo-EM, we solved structures of one or two CD4s bound to the clade A BG505 trimer^14^ to 3.4 and 3.9 Å, respectively. We found that binding one CD4 primarily resulted in a closed, prefusion Env conformation that showed only subtle indications of CD4-induced changes. Binding two CD4 molecules induced an asymmetric, partially open Env conformation in which the gp120 subunits resembled open (for CD4-bound protomers) and occluded-open (for the unliganded protomer) conformations, whereas the three gp41 subunits were structurally different from each other. Together, these results illustrate intermediate Env conformations and inform our understanding of the events that lead to HIV-1 fusion.

## Heterotrimer Env construct design

**Extended Data Fig. 1:**
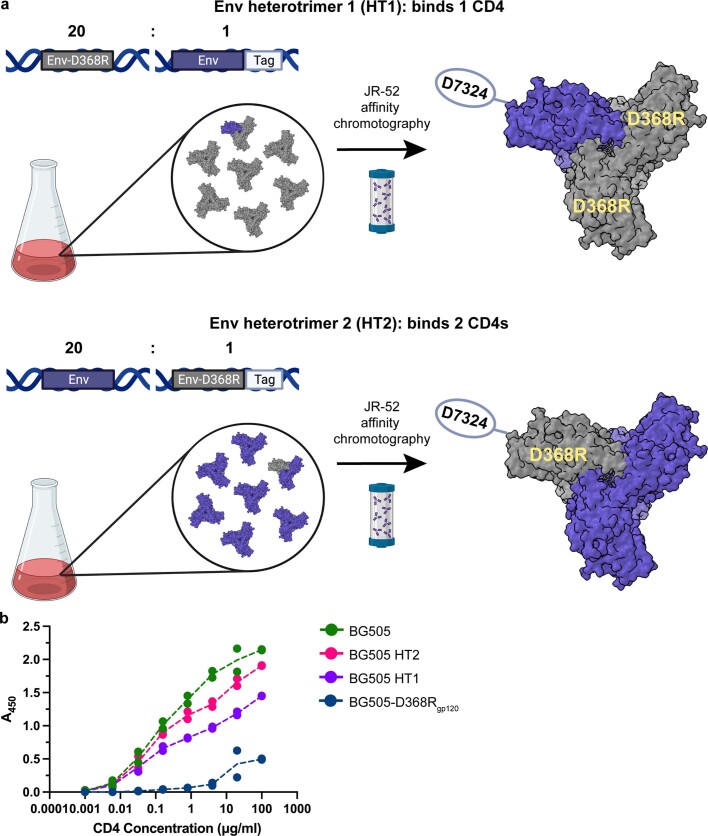
Design and validation of soluble HIV-1 Env heterotrimer constructs. **a**, Methods used to create soluble HT1 and HT2 HIV-1 Env heterotrimers. A 20:1 transfection ratio of untagged and D7324-tagged Env expression plasmids, one of which encoded the D368R CD4 knockout mutation in gp120, was co-transfected to produce two predominant populations: untagged trimers and singly tagged trimers. Transfection supernatants were harvested and Env proteins purified by JR-52 immunoaffinity chromatography (as described), resulting in the HT1 and HT2 heterotrimers. Schematics were generated using BioRender.com. **b**, ELISA comparing CD4 binding of BG505, BG505 HT2, BG505 HT1, and BG505-D368R. Values from two biological replicates (n = 2) are represented by points, with the mean indicated by the dotted line.

A soluble heterotrimer Env that can bind only one CD4 receptor, termed HT1, was generated by coexpressing plasmids encoding BG505 SOSIP.664 (refs. ^14,20^) bearing a D368R_gp120_ mutation that eliminates CD4 binding^21,22^ and an affinity-tagged mutant BG505 at a 20:1 ratio (Extended Data Fig. 1a). For HT2, designed to bind only two CD4 receptors, plasmids encoding BG505 SOSIP.664 and a tagged BG505-D368R_gp120_ SOSIP.664 were coexpressed in a 20:1 ratio (Extended Data Fig. 1a). Assuming random assembly, 13% of the Env population would be the desired singly tagged heterotrimer, and less than 1% would contain dually and triply tagged trimers^23^. For both constructs, immunoaffinity column purification resulted in purified tagged heterotrimers (Extended Data Fig. 1a).

To validate BG505 HT1 and BG505 HT2, we performed enzyme-linked immunosorbent assays (ELISAs) to compare binding of soluble CD4 to heterotrimeric Envs and to homotrimeric wild-type and D368R_gp120_ mutant Envs (Extended Data Fig. 1b). As expected, wild-type BG505 exhibited the highest level of CD4 binding, BG505-D368R_gp120_ showed only limited CD4 binding at high concentrations, and BG505 HT1 and BG505 HT2 showed intermediate levels of CD4 binding, with more binding to BG505 HT2 than to BG505 HT1.

## Closed conformation of one CD4-bound Env

**Fig. 1:**
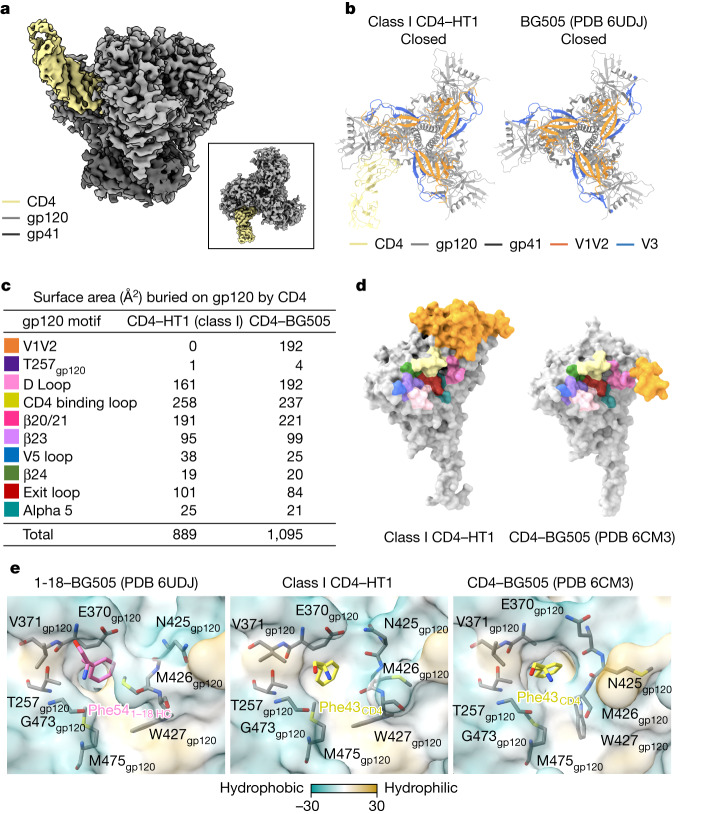
3.4 Å cryo-EM structure of BG505 HT1 bound to one CD4 shows closed, prefusion Env conformation. **a**, Side view of the 3.4 Å class I CD4–HT1 density map. Inset, top-down view. **b**, Top-down cartoon representations of class I CD4–HT1 and BG505 (PDB 6UDJ; 1-18 and 10-1074 antibodies are not shown) structures with gp120 V1V2 and V3 loops highlighted. **c**, Table summarizing BSA on gp120 from CD4 binding for class I CD4–HT1 and CD4–BG505 (PDB 6CM3) complexes. **d**, Surface representation comparisons of class I CD4–HT1 and CD4–BG505 (PDB 6CM3). **e**, Surface representations depicting hydrophobicity (Kyte–Doolittle scale^49^) for 1-18–BG505 (PDB 6UDJ), class I CD4–HT1 and CD4–BG505 (PDB 6CM3) overlaid with stick representations of gp120 residues within the Phe43 cavity.

**Extended Data Fig. 2:**
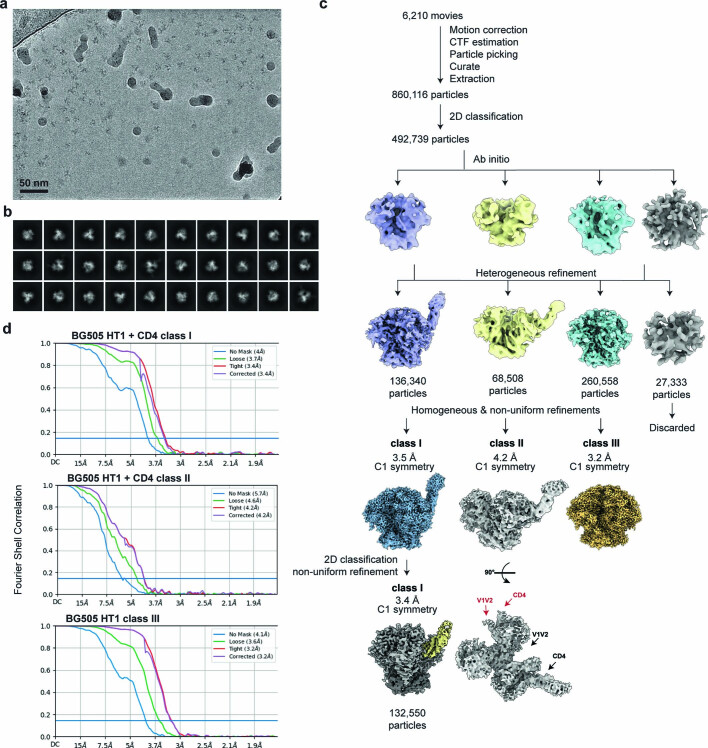
Cryo-EM data processing and validation for BG505 HT1 in complex with CD4. **a**, Representative micrograph and **b**, representative 2D classes for the CD4-BG505 HT1 complex. **c**, Workflow of single-particle cryo-EM data processing. Class I shows one CD4 bound to a closed, prefusion conformation of HT1. Class II shows density for one CD4 molecule bound to HT1 and weak density for an additional bound CD4 (red arrow). The class II density map may represent an average of HT1 Envs bound to one or two CD4 molecules. We were unable to further separate these particles into different subclasses. Class III resembles an unliganded, closed prefusion Env trimer. **d**, Fourier shell correlation (FSC) plots of the final reconstructions for CD4-BG505 HT1 classes I, II, and III.

We used single-particle cryo-EM to solve structures of the BG505 HT1 heterotrimer in the presence of CD4 (Fig. 1a, Extended Data Fig. 2 and Extended Data Table 1). We found three classes of HT1 heterotrimer: class I (132,550 particles; 3.4 Å resolution; density for one bound CD4), class II (68,508 particles; 4.2 Å resolution; strong density for one bound CD4 and weak density for a second bound CD4), and class III (260,558 particles; 3.2 Å resolution; no density corresponding to bound CD4 molecules). Weak density for a second bound CD4 in the class II reconstruction indicates that this map may represent an average of HT1 Envs in different conformations bound to one or two CD4 molecules, with one CD4-bound Envs in the majority. Owing to heterogeneity (class II) or no bound CD4 (class III), we fitted CD4 and Env trimer coordinates to only the class I CD4–HT1 reconstruction.

Despite CD4 recognition of one gp120 protomer, the class I HT1 trimer maintained the prefusion closed Env conformation with V1V2 loops at the apex and V3 loops shielded beneath V1V2 (refs. ^1,2,14^) (Fig. 1b and Supplementary Video 1), indicating that interactions of a soluble Env with one CD4 molecule are predominantly insufficient to trigger conformational changes that lead to trimer opening^5,6,9^. CD4 binding to a closed Env conformation was also observed in a low-resolution structure of CD4 bound to a homotrimeric SOSIP that included mutations to prevent Env opening^24^. However, the HT1 heterotrimer used for the structural studies here did not include mutations that lock Env into a closed, prefusion conformation.

We compared interactions in the CD4bs of the CD4-bound protomer of the class I CD4–HT1 complex with the CD4bs in the gp120 of a CD4-bound open BG505 trimer (PDB 6CM3) by calculating the surface area of gp120 buried by CD4 (buried surface area; BSA) (Fig. 1c,d). In the CD4bs region of gp120, the CD4 BSA footprints were comparable for class I CD4–HT1 and CD4–BG505. However, V1V2 displacement in the CD4–BG505 complex resulted in approximately 200 Å^2^ more BSA on gp120 (Fig. 1c,d). These contacts have been previously demonstrated to stabilize the CD4-induced open conformation of Env^5,9^. Thus, the Env–CD4 interface remained largely unchanged during CD4 engagement with HT1, with the primary difference between one CD4-bound HT1 and CD4-bound open homotrimeric Env structures^5–9^ being displacement of V1V2 to the side of gp120 where it makes further contacts with CD4.

CD4-induced Env conformational changes are triggered, at least in part, by insertion of Phe43_CD4_ into a conserved, hydrophobic cavity (the Phe43 cavity) on gp120 (refs. ^5,6,9,25,26^). Small-molecule CD4 mimetics such as BNM-III-170 and M48U1 insert hydrophobic entities into the Phe43_CD4_ cavity, thereby competing with CD4 binding and inducing Env opening^8,27–32^. Some CD4bs broadly neutralizing antibodies (bNAbs) also mimic Phe43_CD4_ interactions by inserting a hydrophobic residue at antibody heavy chain (HC) position 54 into the Phe43 cavity on gp120. However, in contrast to the conformational effects of CD4 and selected small mimetic inhibitors on Env conformation, CD4bs bNAbs with a hydrophobic HC residue 54 stabilize the prefusion closed Env conformation when bound to trimeric Env^33–37^.

To examine the consequences of insertion of Phe43_CD4_ into a single gp120 Phe43 cavity in the class I CD4–HT1 complex, we compared the structural landscape of the Phe43 cavity in the gp120s of two symmetric Env trimer complexes: the CD4bs bNAb 1-18 bound to a closed, prefusion conformation BG505 (ref. ^35^) and CD4 bound to an open, fully CD4-saturated BG505 trimer^9^ (Fig. 1e). We identified and compared the positions of conserved residues in the Phe43 cavity, some of which undergo rearrangements during CD4-induced Env opening^6,25^. Residues in the CD4 binding loop (E370_gp120_, V371_gp120_) together with T257_gp120_ and exit loop (G473_gp120_, M475_gp120_) residues maintained analogous positions in the class I one CD4-bound HT1, zero CD4-bound closed and three CD4-bound open trimers (Fig. 1e). However, subtle differences in the gp120 β20/β21 loop were observed; for instance, in the 1-18–BG505 complex, the N425_gp120_ side chain pointed away from Phe54_1-18 HC_, whereas the M426_gp120_ side chain pointed towards Phe54_1-18 HC_, and the planes of the W427_gp120_ side chain and Phe54_1-18 HC_ side chain were parallel. By contrast, in the CD4–BG505 open complex, the N425_gp120_ side chain pointed upwards from the Phe43 cavity ceiling, the M426_gp120_ side chain pointed away from Phe43_CD4_ and the W427_gp120_ side chain was perpendicular to the Phe43_CD4_ side chain. The class I CD4–HT1 complex showed an intermediate orientation of gp120 β20/β21 loop residues, with the N425_gp120_ and M426_gp120_ side chains oriented similarly to their positions in the CD4–BG505 complex, whereas the W427_gp120_ side chain adopted a position similar to that in the 1-18–BG505 complex. Thus, whereas the overall conformation of the Env trimer in the class I CD4–HT1 complex represented a closed, prefusion Env, the gp120 Phe43 cavity showed indications of structural changes consistent with CD4 binding.

## Open conformation of two CD4-bound Env

**Extended Data Fig. 3:**
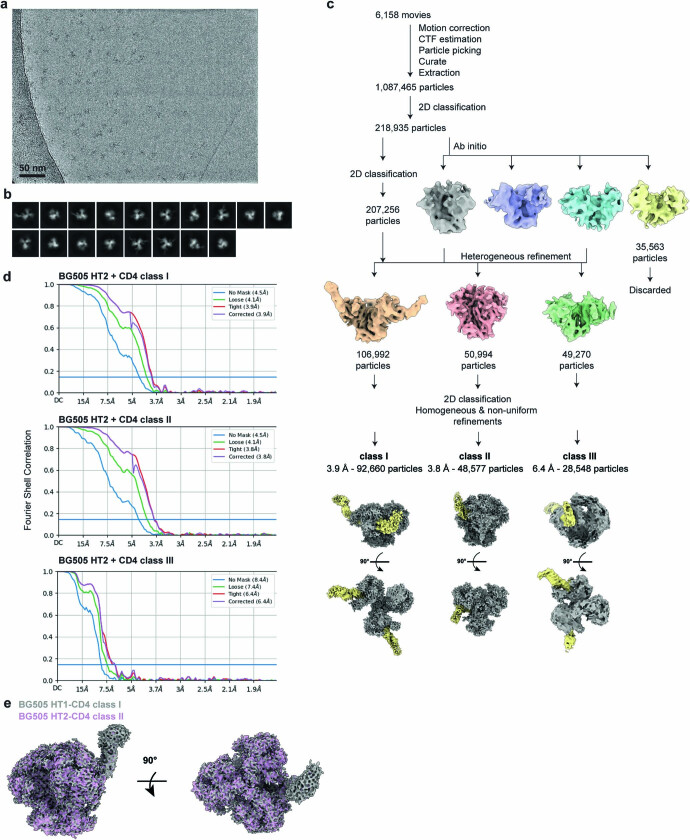
Cryo-EM data processing and validation for BG505 HT2 in complex with CD4. **a**, Representative micrograph and **b**, representative 2D classes for the CD4-BG505 HT2 complex. **c**, Workflow of single-particle cryo-EM data processing. **d**, Fourier shell correlation (FSC) plot of the final reconstruction for CD4-BG505 HT2 classes I, II, and III. **e**, Side and top-down views of superimposed CD4-BG505 HT1 class I and CD4-BG505 HT2 class II single-particle cryo-EM density maps. The densities for CD4 overlap extensively, thus obscuring the purple density for CD4 in the BG505 CD4-HT2 map.

To structurally characterize BG505 HT2 complexed with CD4, we collected single-particle cryo-EM data, recovering three classes (Extended Data Fig. 3 and Extended Data Table 1): class I (92,660 particles; 3.9 Å resolution; BG505 heterotrimer with two CD4-bound protomers and one unliganded protomer), class II (48,577 particles; 3.8 Å resolution, BG505 trimer bound to a single CD4 and similar to the class I CD4–HT1 structure; Extended Data Fig. 3e), class III (28,548 particles; 6.4 Å resolution; poorly resolved with one Env protomer showing clear CD4 density, an adjacent protomer with less defined CD4 density, and density for the third, unliganded protomer extending across the trimer apex to potentially contact the adjacent CD4-bound protomer). Subsequent analyses of the CD4–BG505 HT2 complex were confined to the 3.9 Å class I structure.

**Fig. 2:**
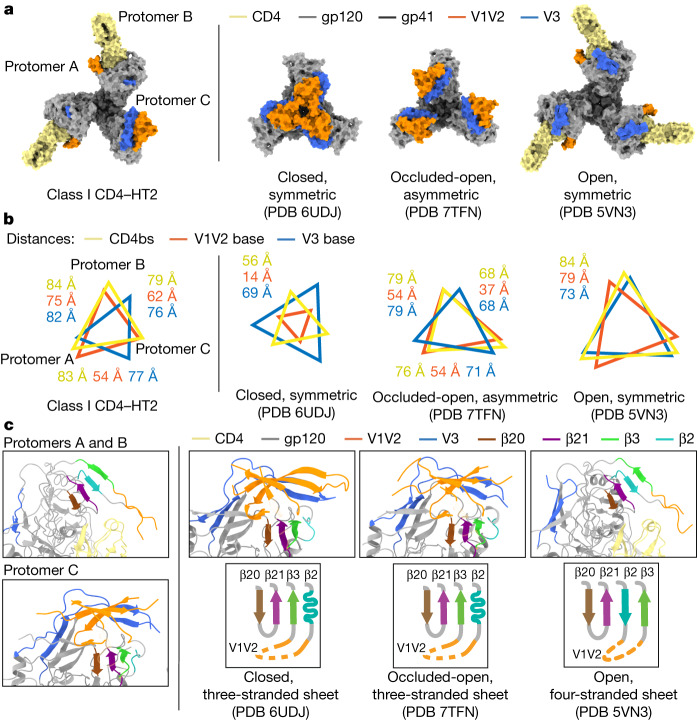
3.9 Å cryo-EM structure of BG505 HT2 bound to two CD4 molecules shows an asymmetric open Env conformation. **a**, Top-down views of surface depictions of class I CD4–HT2, an Env in a prefusion conformation (PDB 6UDJ), Env in the occluded-open conformation (PDB 7TFN) and the CD4-bound open conformation of Env (PDB 5VN3). **b**, Interprotomer distance measurements between reference residues for the base of the V3 loop (H330_gp120_), the base of the V1V2 loop (P124_gp120_) and the CD4bs (D/R368_gp120_) for class I CD4–HT2, an Env in a prefusion conformation (PDB 6UDJ), Env in the occluded-open conformation (PDB 7TFN) and the CD4-bound open conformation of Env (PDB 5VN3). **c**, Cartoon representations of the gp120 bridging sheet motif for class I CD4–HT2, an Env in a prefusion conformation (PDB 6UDJ), Env in an occluded-open conformation (PDB 7TFN) and the CD4-bound open conformation of Env (PDB 5VN3).

We quantified gp120 rearrangements using measurements of interprotomer distances between the Cα atoms of conformationally characteristic Env residues to compare the class I two CD4-bound HT2 structure with other Env conformations (Fig. 2a,b). The relationship between the CD4-bound HT2 gp120 protomers resembled a typical CD4-induced open conformation^5–9^, with V1V2 loops displaced from the Env apex to the sides of gp120 and V3 loops exposed (Fig. 2a), consistent with increased interprotomer distances between these protomers compared with closed^35^, occluded-open^10^ and partially open^9^ Env conformations (Fig. 2b). The unliganded HT2 protomer did not show V1V2 or V3 loop movement to the extent observed in the CD4-bound protomers. Instead, the V1V2 and V3 loops were displaced as a rigid body from the Env apex, as observed in the protomers of the homotrimeric occluded-open Env conformation^10^ (Fig. 2a). Asymmetry of the HT2 Env with two bound CD4s was demonstrated by variable interprotomer distances: the measured distance between the two CD4-bound gp120s (protomers A and B) was consistent with the open, CD4-bound Env conformation, in contrast to distances between the CD4-bound gp120s and the unliganded gp120 (protomer C), which were slightly smaller than distances between CD4-bound gp120s. Thus, the HT2 Env adopted an asymmetric conformation in which the distance to the central trimer axis was smaller in the unliganded protomer than in the CD4-bound protomers (Fig. 2b and Supplementary Video 1).

As a hallmark of CD4-induced gp120 structural changes is the transition of the three-stranded β-sheet to a four-stranded antiparallel bridging sheet^5–7,9^, we next examined the β-sheet conformations in the class I CD4–HT2 complex. The Env β-sheet conformations observed in the CD4–HT2 complex differed: CD4-bound protomers A and B included the four-stranded bridging sheet observed in CD4-bound open Env trimer structures^5,6,9^, whereas the unliganded gp120 in protomer C contained a three-stranded sheet resembling its counterpart gp120s in closed and occluded-open conformations^10,35^ (Fig. 2c). In summary, the binding of two CD4s to BG505 HT2 resulted in an asymmetric and partially open Env trimer composed of two CD4-bound open-conformation gp120s and one unliganded gp120 in an occluded-open conformation.

**Extended Data Fig. 4:**
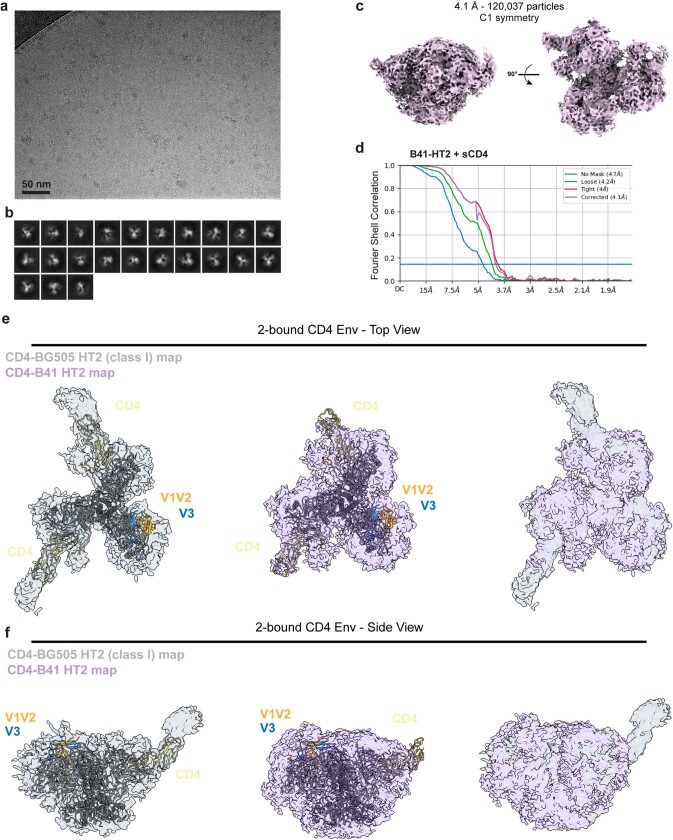
Cryo-EM data processing, validation, and interpretation for B41 HT2 in complex with CD4. **a**, Representative micrograph (5,450 total), **b**, representative 2D classes, and **c**, density map for the CD4-B41 HT2 complex. **d**, Fourier shell correlation (FSC) plot of the final reconstruction for CD4-B41 HT2. **e**,**f**, Top-down (panel e) and side (panel f) views of class I CD4-BG505 HT2 model fit into class I CD4-BG505 HT2 (left) or CD4-B41 HT2 (middle) density maps and alignment of both density maps (right).

To address the generality of these observations, we prepared HT2 heterotrimers for the clade B B41 SOSIP.664 (ref. ^38^) (Extended Data Fig. 1a), obtaining a 4.1 Å cryo-EM density map of B41 HT2 bound to two CD4 molecules (Extended Data Fig. 4). Fitting the CD4–BG505 HT2 structure into the density map for CD4–B41 HT2 showed agreement in overall structural features, including V1V2 displacement of CD4-bound protomers and partial outward gp120 rotation of the unliganded protomer (Extended Data Fig. 4).

**Extended Data Fig. 5:**
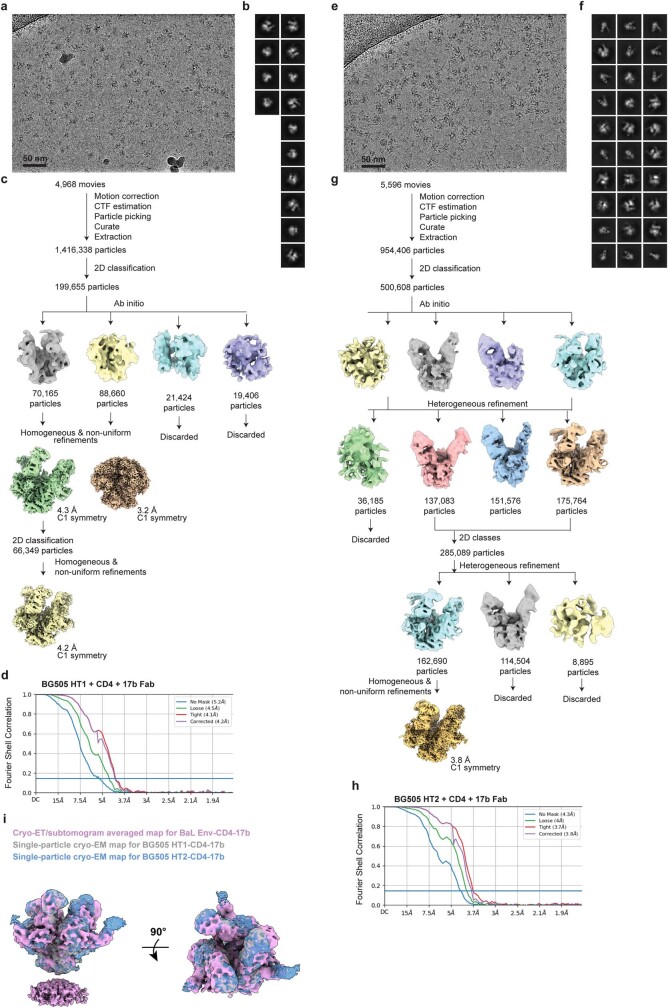
Cryo-EM data processing and validation for BG505 HT1 and BG505 HT2 in complex with CD4 and 17b Fab. **a**, Representative micrograph and **b**, representative 2D classes for the CD4-17b-BG505 HT1 complex. **c**, Workflow of single particle cryo-EM data processing. **d**, Fourier shell correlation (FSC) plot of the final reconstruction for CD4-17b-BG505 HT1. **e**, Representative micrograph and **f**, representative 2D classes for the CD4-17b-BG505 HT2 complex. **g**, Workflow of single particle cryo-EM data processing. **h**, Fourier shell correlation (FSC) plot of the final reconstruction for CD4-17b-BG505 HT2. **i**, Side and top-down views of CD4-17b-BG505 HT1 and CD4-17b-BG505 HT2 single-particle cryo-EM densities (both processed with C1 symmetry) superimposed with the cryo-ET/subtomogram averaged density map (C3 symmetry) of a CD4- and 17b-bound virion-bound BaL Env trimer (EMD-21411). Despite the single-particle cryo-EM structures being derived from heterotrimeric Envs that lack C3 symmetry and the cryo-ET structure being derived from symmetric homotrimeric Envs, the CD4-17b-Env trimer maps are similar.

In addition, we solved 4.2 Å and 3.8 Å structures of CD4 complexes with BG505 HT1 and HT2 plus 17b^39^, a CD4-induced antibody that recognizes the exposed coreceptor binding site on V3 (refs. ^5,6,8,9^) (Extended Data Fig. 5). For both complexes, the Envs showed three bound 17b Fabs and three CD4s and adopted an open conformation, as indicated by density for V1V2 that was displaced to the sides of gp120 on each protomer (Extended Data Fig. 5). Superimposition of CD4–17b–HT1 and CD4–17b–HT2 density maps with a cryo-electron tomography (cryo-ET)/subtomogram-averaged map of membrane-bound BaL Env bound to CD4 and 17b^4^ showed similarities in the orientations of Env gp120s, CD4 molecules and 17b Fabs (Extended Data Fig. 5i). However, poor local map densities surrounding the Fab–gp120 and CD4 interfaces in the single-particle reconstructions with HT1 and HT2 heterotrimers prevented building of reliable atomic models. Although low resolution, these structures can be interpreted by assuming that BG505 Env is in equilibrium between closed and open conformations, with the equilibrium generally favouring the closed, prefusion conformation and transitions to an open conformation in the absence of CD4 binding sampled less frequently. The structural results indicate that the binding of CD4 to an unmutated CD4bs on a gp120 may occur first, enabling subsequent exposure of the V3 loop and binding to 17b Fab in those protomers. Disruption to the prefusion trimer apex through V1V2 displacement probably allows the remaining, unliganded gp120 protomer(s) in the heterotrimer to sample open conformations more frequently, thereby enabling 17b binding. Once the gp120–17b interaction occurs, gp120 could adopt an open conformation with displaced V1V2 loops and become trapped in this state. CD4 could then make contacts with the displaced V1V2, allowing CD4 binding to that protomer. This interaction could overcome the unfavourable effects of the D368R_gp120_ mutation, which would otherwise hinder or prevent CD4 binding to the protomer containing that mutation.

## gp120-mediated changes in gp41

HIV-1 gp41 subunits mediate fusion events between host and viral membranes to enable viral infection^12,40,41^. Prefusion gp41 is composed of a long HR1 helix that extends from beneath the gp120 apex, an HR2 helix that surrounds the amino termini of the HR1 coils and the fusion peptide and fusion peptide proximal region (FPPR) between the HR1 and HR2 helices^12,40,42^. CD4 binding leads to compacting of the carboxyl termini of the HR1 (HR1_c_) helices, triggering formation after coreceptor binding of a prehairpin intermediate in which HR1 extends away from HR2 and the viral membrane^12,40,42^. These movements lead to formation of compact FPPR helices and transitions of the fusion peptides from α-helices shielded in hydrophobic environments to solvent-exposed disordered loops^7,12,40,42^.

**Fig. 3:**
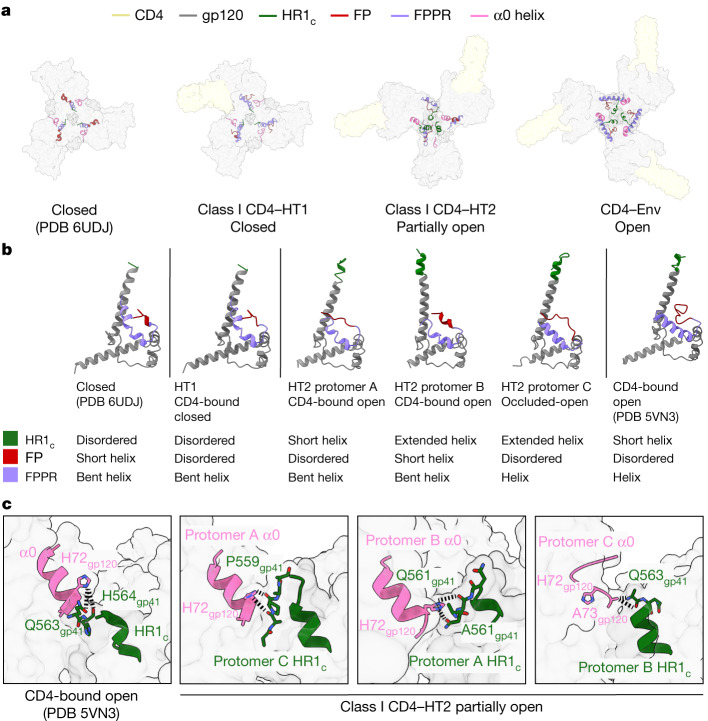
Conformational changes in gp41 were coordinated with gp120 conformation in CD4-bound heterotrimers. **a**, Top-down views of surface representations of Envs from closed (PDB 6UDJ), class I CD4–HT1, class I CD4–HT2 and CD4–Env (PDB 5VN3) structures with gp41 structural elements (HR1_c_, fusion peptide (FP), FPPR, α0 helix) depicted in cartoon representations. **b**, Cartoon representations of gp41 subunits from closed (PDB 6UDJ), class I CD4–HT1, class I CD4–HT2 (protomers A–C) and CD4–Env (PDB 5VN3) structures with coloured gp41 (HR1_c_, FP, FPPR, α0 helix) structural elements. **c**, Cartoon representations of the α0 helix and HR1_c_ for CD4–Env (PDB 5VN3) and class I CD4–HT2 (protomers A–C) with stick representations of selected amino acids. Black dashed connecting lines indicate gp120–gp41 interactions within 6.0 Å.

Previous studies have indicated that changes in Env gp120 conformation may correlate with gp41 changes, indicating cooperativity between the gp120 and gp41 subunits^5–7,9^. Indeed, in closed and CD4-saturated open Env conformations, gp41 subunits undergo the characterized CD4-induced changes described above^5–9^ (Fig. 3a). Closed Env trimers contain gp41s with a disordered HR1_c_, a helical fusion peptide and an FPPR bent helix, whereas the gp41 subunits in open Envs contain a helical HR1_c_, disordered fusion peptide and straight helical FPPR (Fig. 3b). The gp41 subunits in the class I CD4–HT1 complex largely represent gp41s in closed Env trimers, in which each of the three gp120 and gp41 subunits largely retain closed, prefusion conformations despite binding one CD4 (Fig. 3a). The only deviation from the closed gp41 conformation in the class I HT1 heterotrimer is a disordered fusion peptide in all protomers (Fig. 3b).

In the class I CD4–HT2 complex, individual gp41 subunits adopted distinct conformations despite nearly identical conformations of the two CD4-bound gp120s (Fig. 3a,b). The gp41 in CD4-bound protomer A showed a slanted HR1 helix, a short helical HR1_c_, a disordered fusion peptide and a bent helical FPPR (Fig. 3b). The other CD4-bound gp120 in protomer B contained contrasting elements in gp41: the HR1 and HR1_c_ helices were erect (HR1) or fully extended (HR1_c_), consistent with CD4-induced structural changes (Fig. 3b). By contrast, the fusion peptide and FPPR resembled their conformations in closed Envs (Fig. 3b). Despite protomer C being unliganded, its gp41 most resembled the CD4-induced gp41 conformation, with a helical HR1_c_, disordered fusion peptide and a helical FPPR (Fig. 3b). Thus, individual gp41 subunits can adopt different, distinct conformations in the context of a two CD4-bound Env.

A potential link between gp120 and gp41 Env conformations involves the gp120 α0 region. During Env trimer opening, the HR1_c_ extension displaces the α0 disordered loop located above HR1_c_ in the prefusion conformation and forms a stable α-helix that caps the neighbouring gp41 HR1 helix^5,7,8^ (Fig. 3c). In the class I CD4–HT1 complex, the α0 loops resembled those in the prefusion conformation, whereas the α0 conformations in the CD4–HT2 complex were variable (Fig. 3a,c). Despite only a partial extension of HR1_c_ in CD4-bound protomer A of the class I HT2 heterotrimer, the gp120 α0 helix was formed and displaced towards the protomer C HR1_c_, where it was stabilized through interactions with the short disordered protomer C HR1_c_ tip (Fig. 3c). Similarly, for CD4-bound protomer B, HR1_c_ extension created a gp120 α0 helix that interacted with its neighbouring protomer A HR1_c_ (Fig. 3c). In unliganded protomer C, the gp120 α0 region remained in the prefusion disordered loop conformation despite extension of its HR1_c_ (Fig. 3c). The loop conformation was probably accommodated because protomer C gp120 does not undergo the full outwards displacement from the Env trimer axis. However, partial outwards rotation of the protomer C gp120 still enabled interactions with the neighbouring protomer B HR1_c_ (Fig. 3c). These interprotomer interactions between gp120s and gp41s in CD4–HT2 rationalize why each gp41 subunit adopted a distinct conformation, indicating that formation of the α0 helix may be dependent on CD4 occupancy and probably drives gp41 conformational changes.

## CD4-bound SOSIP and virion Envs

In a study described in an accompanying paper, cryo-ET and subtomogram averaging was used to determine the conformations of membrane-bound Envs complexed with substoichiometric numbers of membrane-bound CD4s^11^, allowing comparison of our higher resolution soluble CD4–soluble heterotrimer Env structures with structures of CD4–Env complexes investigated under more physiological conditions.

**Extended Data Fig. 6:**
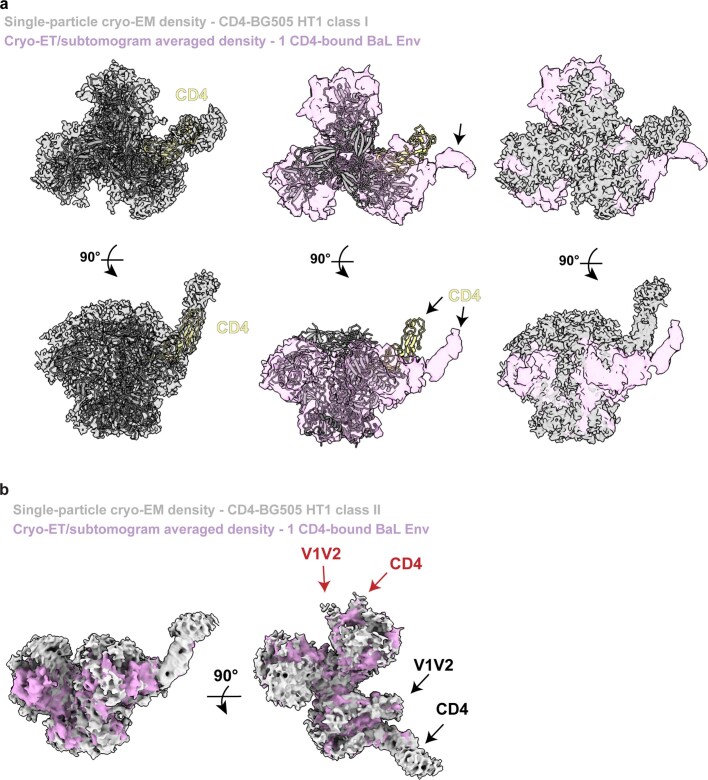
Comparisons of single particle cryo-EM densities of CD4-HT1 with one CD4-bound Env cryo-ET/subtomogram averaged density. **a**, Top-down (top) and side (bottom) views of class I CD4-HT1 model fit into one-CD4 bound Env from single-particle cryo-EM (left) or cryo-ET/subtomogram averaged (middle) density maps. Right: alignment of single-particle cryo-EM and cryo-ET/subtomogram averaged density maps. **b**, Side and top-down views of class II CD4-HT1 single-particle cryo-EM density superimposed with one CD4-bound Env from cryo-ET/subtomogram averaged density map. The densities for CD4 overlap extensively, thus obscuring the purple density for CD4 in the cryo-ET map. Weak densities in the single-particle cryo-EM map for a second bound CD4 and a rearranged V1V2 in the protomer adjacent to the protomer with strong CD4 density are marked with red arrows.

Rigid-body fitting of the class I CD4–HT1 model into the cryo-ET/subtomogram-averaged density of a one CD4-bound Env trimer showed substantial differences (Extended Data Fig. 6a). Unlike the closed Env conformation observed for the soluble class I CD4–HT1 complex (Fig. 1a), the membrane-bound Env adopted a partially open conformation in response to engagement with a single CD4 in which the CD4-bound protomer seemed to undergo CD4-induced conformational changes consistent with V1V2 displacement^11^ (Extended Data Fig. 6a). However, the single-particle cryo-EM-derived heterogeneous class II CD4–HT1 complex reconstruction (Extended Data Fig. 2c) superimposed well with the cryo-ET/subtomogram-averaged density for the one CD4-bound Env trimer^11^ (Extended Data Fig. 6b), consistent with the ability of soluble and membrane-bound Envs to adopt similar conformations on binding of a single CD4.

**Fig. 4:**
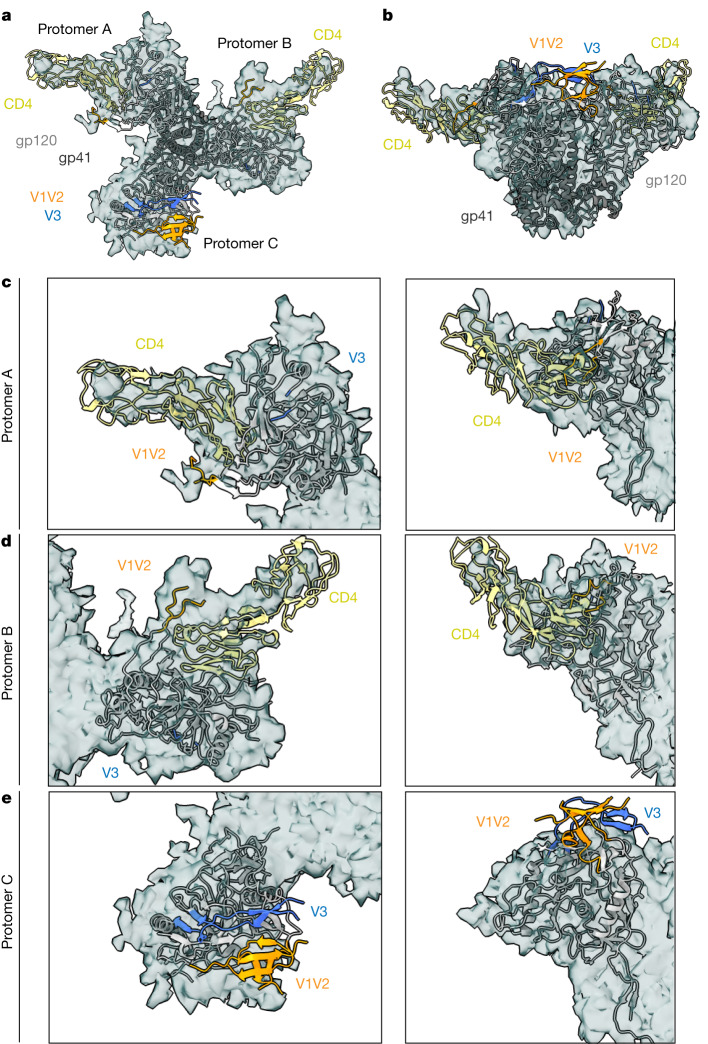
The CD4–HT2 heterotrimer resembles a two CD4-bound membrane-bound Env. **a**,**b**, Top-down (**a**) and side (**b**) views of a cartoon representation of the class I CD4–HT2 structure fitted into density (grey surface) of a two CD4-bound Env derived from cryo-ET/subtomogram averaging^11^. **c**–**e**, Close-ups of protomer A (**c**), protomer B (**d**) and protomer C (**e**) from top-down and side views depicted in **a** and **b**, respectively.

The two CD4-bound membrane-embedded and soluble Envs exhibited similar conformations. Rigid-body fitting of the soluble class I CD4–HT2 structure into the corresponding cryo-ET/subtomogram-averaged CD4–Env density showed alignment of bound CD4s and Env gp120s (Fig. 4a,b). The displaced V1V2 loops in the CD4–HT2 CD4-bound protomers A and B were clearly matched with density from membrane-embedded Env (Fig. 4c,d), and the partial outward gp120 rotation described in unliganded protomer C in the soluble CD4–Env structure (Fig. 2a,b) aligned with density for the unliganded protomer in the membrane-bound Env (Fig. 4e). However, the V1V2 and V3 densities were not resolved in the cryo-ET map^11^, probably owing to flexibility of this region, limiting our comparisons of the V1V2 and V3 regions of the unliganded protomer in membrane-bound Env and soluble Env (Fig. 4e).

## Discussion

HIV-1 Env trimers on virions are likely to encounter multiple CD4 receptors on the surfaces of target cells. However, experimental studies have yet to definitively address whether one, two or all three CD4bs on each trimer must be occupied to induce characterized structural rearrangements in Env (for example, V1V2 displacement and gp120 rotation) that expose the coreceptor binding site. In addition, the degree of cooperativity between Env protomers on binding to CD4 have not been investigated structurally. The characterization of a non-neutralizing antibody isolated from an immunized macaque that mimicked fusion peptide interactions with a single gp41 per trimer, rendering one fusion peptide per trimer inactive^43^, implies that not all protomers in each Env trimer are required for virus–host cell membrane fusion. Consistent with this conclusion, fusion and infectivity studies that incorporated Env mutations resulting in defective CD4, coreceptor and fusion activity in individual protomers of Env heterotrimers^44–47^ indicated that Env entry may not require each subunit in an individual trimer to be competent in performing all functions^44–46^. However, the effects of substoichiometric binding of CD4 in these experiments were complicated by the necessity for Env protomers with different defective mutations to be randomly assembled as homotrimeric and heterotrimer Envs that were compared for fusion and infectivity with homotrimeric controls^44–46^. In addition, these experiments did not include structural characterizations to examine the conformational effects of substoichiometric CD4 interactions with individual Env trimers. Our single-particle cryo-EM investigation of Env heterotrimers binding one or two CD4s, together with the accompanying cryo-ET visualization of the native HIV-1 virions and membrane-bound CD4 (ref. ^11^), adds to our knowledge of Env structures, which was previously limited to closed, prefusion Env conformations with either no bound CD4s or three CD4s bound to fully saturated open Env trimers^1–6,8,9,11^.

By engineering soluble Env heterotrimers with either one or two wild-type CD4bs, we solved structures of Env trimers with substoichiometric numbers of bound CD4s at sufficient resolutions to monitor CD4-induced changes to gp120 and gp41 subunits. We found that binding of one CD4 to the dominant class I three-dimensional reconstruction of CD4–HT1 resulted in minor structural changes to a native-like soluble Env trimer in the closed, prefusion state; for example, we did not observe opening of any of the gp120 subunits of the trimer or the accompanying changes in the CD4-bound gp120 that result from CD4 associating with gp120 in CD4-bound open trimers^5–9^ (in particular, changes resulting from insertion of Phe43_CD4_ into a gp120 hydrophobic cavity, which facilitates induced changes such as V1V2 displacement in CD4-bound gp120 subunits of fully saturated open Env trimers^5,6,9,25,26^, were minor). By contrast, the one CD4-bound conformation of the membrane-bound Env trimer revealed by cryo-ET/subtomogram averaging showed a partially open conformation in which the CD4-bound protomer seemed to undergo CD4-induced conformational changes^11^. This conformation aligned well with a second CD4–HT1 cryo-EM reconstruction, a subdominant heterogeneous class that also showed partial Env opening.

**Fig. 5:**
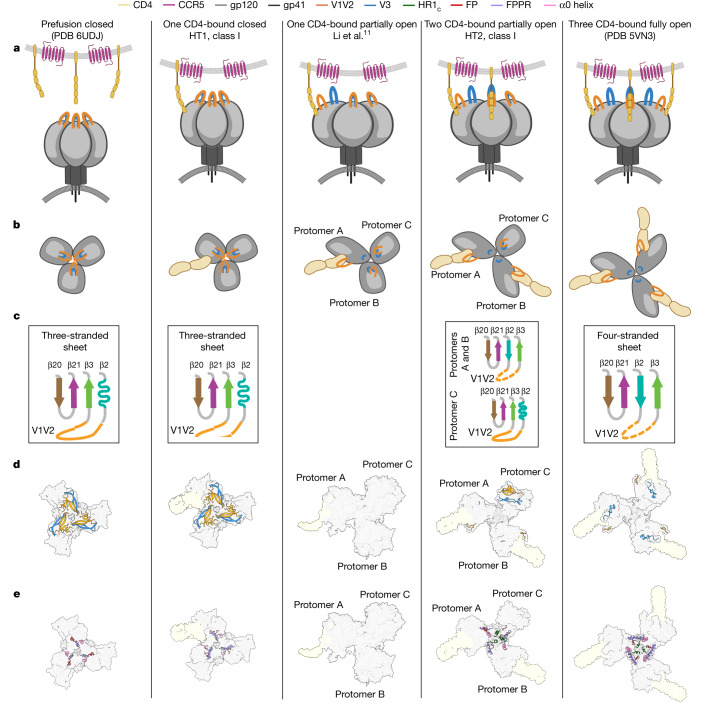
Overview of Env receptor-induced conformational changes that lead to coreceptor binding and fusion. **a**–**e**, Summary of Env conformational changes, including Envs in prefusion closed (PDB 6UDJ), one CD4-bound closed (class I CD4–HT1), one CD4-bound open (cryo-ET)^11^, two CD4-bound partially open (class I CD4–HT2) and three CD4-bound (PDB 5VN3) conformations. The class II CD4–HT1 cryo-EM reconstruction was similar to the partially open Env conformation observed by cryo-ET for one CD4-bound Env^11^, but coordinates were not modelled owing to heterogeneity. Depictions for each Env conformation include: Env schematics in side (**a**) and top-down (**b**) views, diagrams describing β-sheet conformations observed in Env gp120s (**c**), surface representations of structures for each Env conformation with cartoon representations of V1V2 and V3 loops (**d**) and gp41 structural features (**e**). **a** and **b** created using BioRender.com.

The single-particle cryo-EM class I CD4–HT1 and the cryo-ET structures of one CD4-bound Env trimers may represent different conformational intermediates involved in engagement of a single CD4, with the closed trimer conformation likely to precede the more open conformation (Fig. 5). Several factors could contribute to the observation of these different one CD4-bound Env trimer conformations: (1) differences in the Env clade being investigated (tier 2 BG505 for single-particle cryo-EM versus tier 1B BaL for cryo-ET), with tier 2 viruses being more resistant than tier 1 to neutralization and probably also CD4-induced changes^48^; (2) the increased ability of membrane-bound CD4 compared with soluble CD4 to engage with and then dissociate from Envs over the course of an incubation, perhaps leading to visualization in the cryo-ET experiments of one CD4-bound Envs that had recently bound two CD4s; (3) SOSIP substitutions that stabilize the prefusion, closed conformation (including the interprotomer disulfide, I556P, A316W)^14,20^ preventing CD4-induced structural changes when only one CD4 is bound; (4) a lower temperature incubation for the single-particle cryo-EM CD4–HT1 complex (4 °C) compared with the analogous cryo-ET sample (room temperature), perhaps contributing to observation of the closed trimer conformational state with one CD4 bound that probably precedes a more open trimer conformation (Fig. 5). In any case, the heterogeneous class II CD4–HT1 single-particle cryo-EM reconstruction superimposed well with the one CD4-bound cryo-ET Env density (Extended Data Fig. 6b), indicating the ability of the SOSIP HT1 Env to adopt a more open conformation in response to primarily binding a single CD4.

The single-particle cryo-EM and cryo-ET structures of two CD4-bound Env trimers were remarkably consistent, such that both showed two protomers in CD4-bound open conformations and the remaining unbound protomer in a conformation resembling an occluded-open Env protomer^10^ (Fig. 4). These results provide further evidence of SOSIP Env trimers resembling their virion-bound counterparts^3,4^, both in the closed, prefusion conformation and in various CD4-bound conformations that adopt different conformations compared with unliganded Env trimers. Thus, this study and the accompanying cryo-ET imaging^11^, together with previous Env structures, complete a description of the conformations of HIV-1 Env trimers at each stage of engaging CD4, from no bound receptors to the final conformation with three bound receptors (Fig. 5 and Supplementary Video 1).

The ability to confirm single-particle soluble Env heterotrimer conformations that include residue-level details using lower-resolution Env trimer structures derived by cryo-ET under more physiological conditions^11^ lends confidence to the proposed order of structural transitions induced by CD4 binding (Fig. 5 and Supplementary Video 1). The single-particle cryo-EM structures also include descriptions of details of CD4-induced structural changes in gp120 and gp41, including cooperative intersubunit structural transitions. These results reveal intermediate Env conformations that expand our understanding of receptor-induced structural changes preceding host and viral membrane fusion, thereby informing the design of therapeutics to block HIV-1 infection.

## Methods

### Protein expression and purification

SOSIP.664v4 Env constructs included the following stabilizing mutations: introduced cysteines 501C and 605C, I559P, A316W and the furin cleavage site mutated to six arginine residues^14,20^. SOSIPs with D7324 tags included a GSAPTKAKRRVVQREKR sequence after residue 664 in the gp41 ectodomain^14^. The D368R mutation was encoded in Envs to impair CD4 binding^21,50–52^. Genes encoding tagged and untagged SOSIP.664 Env homotrimers were expressed by transient transfection of Expi293 cells (Thermo Fisher Scientific). Env heterotrimers were purified from cotransfections involving a 20:1 expression plasmid DNA ratio of untagged to tagged Env constructs: a 20:1 ratio of Env-D368R/Env-D7324 (HT1) and a 20:1 plasmid of Env/Env-D368R-D7324 (HT2). Trimeric Envs were purified from cell supernatants by PGT145 immunoaffinity chromatography and size-exclusion chromatography (SEC) using a Superose 6 10/300 column (Cytiva)^14,53^. Tagged Env homotrimers and heterotrimers (HT1 and HT2) were further purified using JR-52 immunoaffinity chromatography as previously described^14^.

Genes encoding CD4 D1D2 (domains 1 and 2) and D1–D4 (domains 1–4) with C-terminal 6x-His or StrepII tags were transiently transfected using the Expi293 expression system (Thermo Fisher Scientific)^6^. CD4 proteins were purified using Ni^2+^-NTA (Cytiva) or Strep-Tactin XT (IBA Life Sciences) affinity columns, followed by SEC using a Superdex 200 10/300 column (Cytiva).

The Fab from the CD4i antibody 17b^39^ was expressed by transient transfection using expression vectors encoding the light chain and a C-terminally tagged HC portion of the Fab using the Expi293 expression system (Thermo Fisher Scientific)^6^. Fab was purified from cell supernatants by Ni^2+^-NTA (Cytiva) chromatography followed by SEC using a Superdex 200 10/300 column (Cytiva).

### D7324 capture ELISA

ELISAs were performed as previously described^8,10,54^. Briefly, 5 µg/ml of JR-52 IgG^14^ (gift from J. Robinson, Tulane University) was coated on Corning Costar high-binding 96-well plates in 0.1 M NaHCO_3_ (pH 9.6). Plates were incubated overnight at 4 °C. After washing, plates were blocked with 3% bovine serum albumin in TBS-T (20 mM Tris, 150 mM NaCl, 0.1% Tween-20) for 1 h at room temperature. Blocking buffer was removed, and D7324-tagged Envs were applied to plates at 5 µg/ml in 3% bovine serum albumin in TBS-T. Plates were incubated for 1 h at room temperature, and then buffer was removed. For some experiments, 6x-His tagged CD4 was serially diluted in 3% bovine serum albumin in TBS-T at a top concentration of 100 µg/ml and added to plates, followed by incubation for 4 h at room temperature. The CD4 solution was removed, and plates were washed twice with TBS-T. A horseradish-peroxidase-labelled secondary against the His tag (Genscript) was added at a 1:5,000 dilution in 3% bovine serum albumin in TBS-T. Plates were incubated for 30 min and then washed with TBS-T three times. Colorimetric detection of CD4 binding was accomplished using Ultra TMB-ELISA Substrate Solution (Thermo Fisher Scientific), and quenching was performed with 1.0 N HCl. Absorption was measured at 450 nm. Two independent biological replicates (*n* = 2) were used for all assays.

### Assembly of protein complexes and cryo-EM sample preparation

The D1–D4 version of CD4 was chosen instead of CD4 D1D2 for structural studies with BG505 HT1 and HT2 to increase particle size. HT1–CD4 and HT2–CD4 complexes were prepared by incubating purified Env heterotrimers with a 1.1× molar excess of CD4 D1–D4 overnight at 4 °C. We attempted CD4–Env incubations at different temperatures (namely 37 °C and room temperature) and found that overnight incubation at 4 °C produced the most favourable particle quality when frozen on cryo-EM grids. For HT1–CD4–17b and HT2–CD4–17b complexes, 17b Fab was added before grid-freezing at a 1.1× molar excess, followed by incubation at 4 °C for 30 min. QuantiFoil 300 mesh 1.2/1.3 grids (Electron Microscopy Sciences) were glow discharged with PELCO easiGLOW (Ted Pella) for 1 min at 20 mA. Fluorinated octylmaltoside solution (Anatrace) was added to the protein complex to a final concentration of 0.02% (w/v), and 3 µl of the complex–detergent mixture was applied to glow-discharged grids. A Mark IV Vitrobot (Thermo Fisher Scientific) was used to blot grids for 3 s with 0 blot force using Whatman No.1 filter paper and 100% humidity at room temperature. Grids were plunge-frozen and vitrified in 100% liquid ethane.

### Cryo-EM sample preparation and data collection

Single-particle cryo-EM datasets for HT1–CD4, HT2–CD4, HT1–CD4–17b and HT2–CD4–17b were collected on a 300 keV Titan Krios (Thermo Fisher Scientific) cryo-electron microscope equipped with a K3 direct electron detector camera (Gatan) using SerialEM v.3.7 (ref. ^55^) automated data collection software. Videos were recorded with 40 frames at a total dosage of 60 e^−^/Å^2^ using a 3 × 3 beam image shift pattern with three exposures per hole in super resolution mode, a defocus range of −1 to −3 µm and pixel size of 0.416 Å.

Data were processed using cryoSPARC v.3.2 (ref. ^56^). Patch motion correction was applied to each dataset with a binning factor of 2, followed by Patch CTF to estimate contrast transfer function parameters. The blob picker with a diameter of 100 to 230 Å was used to pick particles. Particles were extracted and then two-dimensional classified. Particle classes representing the expected complex were selected and used for ab initio modelling. The ab initio models and corresponding particles that represented the expected complex underwent subsequent rounds of heterogeneous, homogeneous and non-uniform refinements. Resolutions were calculated in cryoSPARC v.3.2 (ref. ^56^) using the gold-standard Fourier shell correlation 0.143 criterion. Fourier shell correlation plots were generated with cryoSPARC v.3.2 (ref. ^56^).

### Model building and refinement of cryo-EM structures

The model coordinates for class I BG505 HT1–CD4 were generated by fitting the following reference coordinate files into cryo-EM density using UCSF ChimeraX v.1.2.5 (ref. ^57^): BG505 gp120 monomer (PDB 6UDJ), gp41 monomer (PDB 6UDJ) and CD4 D1D2 (PDB 5U1F). For the class I BG505 HT2–CD4 reconstruction, the initial coordinates included the BG505 gp120 CD4-bound monomer (PDB 7LOK), BG505 gp120 unliganded monomer (PDB 7TFN), gp41 monomer (PDB 6UDJ) and CD4 D1D2 (PDB 5VN3). Domains 3 and 4 of CD4 D1–D4 were not modelled owing to potential flexibility between CD4 domains 2 and 3. Initial BG505 HT–CD4 models and *N*-linked glycans were manually refined using Coot v.0.8.9.1 (ref. ^58^). Iterative rounds of whole-complex refinements using Phenix v.1.17.1 (phenix.real_space_refine)^59,60^ and Coot v.0.8.9.1 (ref. ^58^) were then performed to generate the final models.

### Structural analyses

Structure figures were created with PyMOL v.2.4.0 (Schrödinger LLC) and UCSF ChimeraX v.1.2.5 (ref. ^57^). BSA was calculated using PDBePISA^61^ with a 1.4 Å probe. gp120 BSA was calculated for protein components of gp120 without including glycan coordinates. Owing to the low resolution of complexes, interactions were assigned tentatively using the following criteria: hydrogen bonds were assigned as pairwise interactions less than 6.0 Å and with an A-D-H angle greater than 90°, and van der Waals interactions were assigned as distances between atoms that were less than 6.0 Å.

### Reporting summary

Further information on research design is available in the Nature Portfolio Reporting Summary linked to this article.

## Online content

Any methods, additional references, Nature Portfolio reporting summaries, source data, extended data, supplementary information, acknowledgements, peer review information; details of author contributions and competing interests; and statements of data and code availability are available at 10.1038/s41586-023-06639-8.

### Supplementary information

Supplementary Video 1 Conformational states of HIV-1 Env trimer. Summary of CD4-induced conformational changes in Env trimer that occur on binding of one, two and three CD4 receptors.

Reporting Summary

## Figures and Tables

**Extended Data Table 1:**
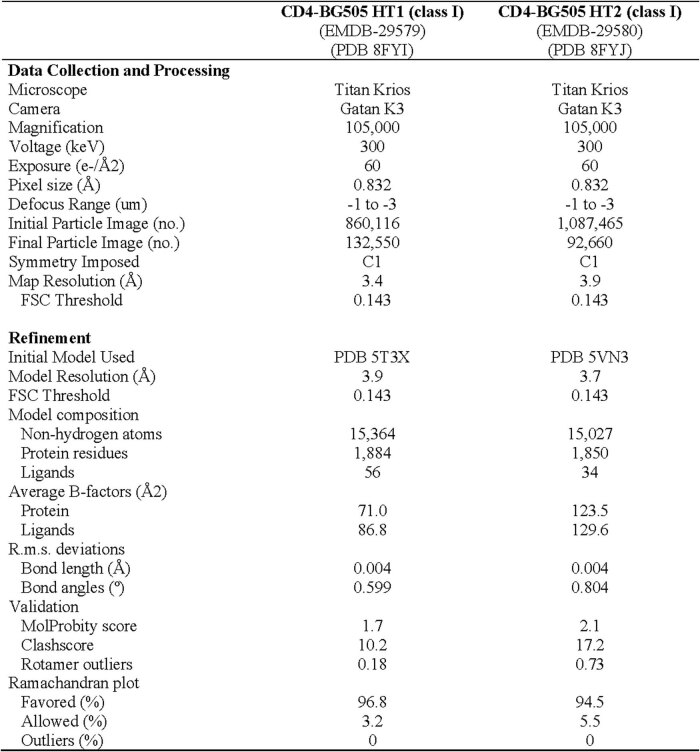
Cryo-EM data collection, refinement, and validation statistics

Single-particle cryo-EM data collection and structure refinement statistics for CD4–BG505 HT1 (class I) (left) and CD4–BG505 HT2 (class I) (right).

## Data Availability

The cryo-EM maps and atomic structures have been deposited in the Protein Data Bank (PDB) and/or Electron Microscopy Data Bank (EMDB) under accession codes 8FYI and EMD-29579 for class I CD4–BG505 HT1, EMD-40437 for class II CD4–BG505 HT1, EMD-40438 for class III BG505 HT1, 8FYJ and EMD-29580 for class I CD4–BG505 HT2, EMD-29581 for class II CD4–BG505 HT2, EMD-29582 for class III CD4–BG505 HT2, EMD-29601 for CD4–B41 HT2, EMD-29583 for CD4–17b–BG505 HT1 and EMD-29584 for CD4–17b–BG505 HT2. PDB entries (6UDJ, 5U1F, 7LOK, 7TFN, 5VN3 and 6CM3) used in this study were downloaded from the PDB. EMDB entry EMD-21411 used in this study was downloaded from the EMDB.
