# Publisher Correction: Intermediate conformations of CD4-bound HIV-1 Env heterotrimers

**DOI:** 10.1038/s41586-024-07082-z

**Published:** 2024-01-19

**Authors:** Kim-Marie A. Dam, Chengcheng Fan, Zhi Yang, Pamela J. Bjorkman

**Affiliations:** 1 Division of Biology and Biological Engineering, California Institute of Technology, Pasadena, CA USA; 2 Present Address: Department of Molecular and Cell Biology, University of California, Berkeley, CA USA

**Keywords:** Cryoelectron microscopy, Virus structures

Correction to: *Nature* 10.1038/s41586-023-06639-8 Published online 22 November 2023

In the version of the article initially published, reference 11 included a preprint title and DOI, and has now been amended to reflect the published article (Li, W. et al. HIV-1 Env trimers asymmetrically engage CD4 receptors in membranes. *Nature* **623**, 1026–1033 (2023)) in the HTML and PDF versions of the article.

