# Supplementary material for: Intermediate conformations of CD4-bound HIV-1 Env heterotrimers
Source: Nature. 2023 Nov 22;623(7989):1017–25. doi: 10.1038/s41586-023-06639-8 (PMC10686819; doi:10.1038/s41586-023-06639-8)
Supplement: Supplementary file 2 — Reporting Summary [file 41586_2023_6639_MOESM2_ESM.pdf]

nature portfolio

Corresponding author(s): Pamela J. Bjorkman
Last updated by author(s): Feb 9, 2023

Reporting Summary

Nature Portfolio wishes to improve the reproducibility of the work that we publish. This form provides structure for consistency and transparency in reporting. For further information on Nature Portfolio policies, see our Editorial Policies and the Editorial Policy Checklist.

Statistics

For all statistical analyses, confirm that the following items are present in the figure legend, table legend, main text, or Methods section.

n/a	Confirmed	
☐	☒	The exact sample size (n) for each experimental group/condition, given as a discrete number and unit of measurement
☐	☒	A statement on whether measurements were taken from distinct samples or whether the same sample was measured repeatedly
☒	☐	The statistical test(s) used AND whether they are one- or two-sided<br>*Only common tests should be described solely by name; describe more complex techniques in the Methods section.*
☒	☐	A description of all covariates tested
☒	☐	A description of any assumptions or corrections, such as tests of normality and adjustment for multiple comparisons
☐	☒	A full description of the statistical parameters including central tendency (e.g. means) or other basic estimates (e.g. regression coefficient) AND variation (e.g. standard deviation) or associated estimates of uncertainty (e.g. confidence intervals)
☒	☐	For null hypothesis testing, the test statistic (e.g. F, t, r) with confidence intervals, effect sizes, degrees of freedom and P value noted<br>*Give P values as exact values whenever suitable.*
☒	☐	For Bayesian analysis, information on the choice of priors and Markov chain Monte Carlo settings
☒	☐	For hierarchical and complex designs, identification of the appropriate level for tests and full reporting of outcomes
☒	☐	Estimates of effect sizes (e.g. Cohen's d, Pearson's r), indicating how they were calculated

Our web collection on statistics for biologists contains articles on many of the points above.

Software and code

Policy information about availability of computer code

Data collection	Data for single-particle cryo-EM were collected on a Titan Krios transmission electron microscope, operating at 300 kV. Movies were recorded with 40 frames at a total dosage of 60 e-/Å2 using a 3×3 beam image shift pattern with 3 exposures per hole in the super resolution mode, a defocus range of −1 to −3 µm, and pixel size of 0.416 Å.
Data analysis	Cryo-EM data were processed using cryoSPARC. Patch motion correction was applied to each dataset with a binning factor of 2, followed by Patch CTF to estimate CTF parameters. The blob picker with a diameter of 100 to 230 Å was used to pick particles. Particles were extracted and then 2D classified. Particle classes representing the expected complex were selected and used for ab initio modeling. The ab initio models and corresponding particles that represented the expected complex underwent subsequent rounds of heterogeneous, homogeneous, and non-uniform refinement.<br><br>Structure figures were created with PyMol (Schrödinger LLC) and UCSF ChimeraX. BSA was calculated using PDBePISA using a 1.4 Å probe. gp120 BSA was calculated for protein components of gp120 without including glycan coordinates. Due to the low resolution of complexes, interactions were assigned tentatively using the following criteria: hydrogen bonds were assigned as pairwise interactions less than 6.0 Å and with an A-D-H angle >90°, and van der Waals interactions were assigned as distances between atoms that were less than 6.0 Å.

For manuscripts utilizing custom algorithms or software that are central to the research but not yet described in published literature, software must be made available to editors and reviewers. We strongly encourage code deposition in a community repository (e.g. GitHub). See the Nature Portfolio guidelines for submitting code & software for further information.

Data

Policy information about availability of data

All manuscripts must include a data availability statement. This statement should provide the following information, where applicable:

- Accession codes, unique identifiers, or web links for publicly available datasets
- A description of any restrictions on data availability
- For clinical datasets or third party data, please ensure that the statement adheres to our policy

The cryo-EM maps and atomic structures have been deposited in the PDB and/or Electron Microscopy Data Bank (EMDB) under accession codes 8FYI [http://doi.org/ 10.2210/pdb8fyi/pdb] and EMD- 29579 [https://www.ebi.ac.uk/pdbe/entry/emdb/EMD-29579] for CD4-BG505 HT1, 8FYJ [http://doi.org/ 10.2210/pdb8fyj/pdb] and EMD-29580 [https://www.ebi.ac.uk/pdbe/entry/emdb/EMD-29580] for CD4-BG505 HT2 (class I), EMD- 29581 [https://www.ebi.ac.uk/pdbe/entry/emdb/EMD-29581] for CD4-BG505 HT2 (class II), EMD- 29582 [https://www.ebi.ac.uk/pdbe/entry/emdb/EMD-29582] for CD4-BG505 HT2 (class III), EMD-29601 [https://www.ebi.ac.uk/pdbe/entry/emdb/EMD-29601] for CD4-B41 HT2, EMD-29583 [https://www.ebi.ac.uk/pdbe/entry/emdb/EMD-29583] for CD4-17b-BG505 HT1, and EMD-29584 [https://www.ebi.ac.uk/pdbe/entry/emdb/EMD-29584] for CD4-17b-BG505 HT2.

Human research participants

Policy information about studies involving human research participants and Sex and Gender in Research.

Reporting on sex and gender	N/A
Population characteristics	N/A
Recruitment	N/A
Ethics oversight	N/A

Note that full information on the approval of the study protocol must also be provided in the manuscript.

Field-specific reporting

Please select the one below that is the best fit for your research. If you are not sure, read the appropriate sections before making your selection.

☒ Life sciences ☐ Behavioural & social sciences ☐ Ecological, evolutionary & environmental sciences

For a reference copy of the document with all sections, see nature.com/documents/nr-reporting-summary-flat.pdf

Life sciences study design

All studies must disclose on these points even when the disclosure is negative.

Sample size	Sample sizes and protein concentrations for cryo-EM were estimated based on previous literatures and have been shown to be sufficient. Samples sizes for ELISA experiments were estimated for biological duplicates.
Data exclusions	Cyro-EM particle images with poor ice quality were excluded during data processing such as 2D classifications and 3D classifications to generate high-resolution EM maps, the method has been proven to be effective by numerous publications.
Replication	ELISA experiments were performed with n=2 independent biological replicates. Cryo-EM data were recorded and processed multiple times and showed same results with different resolutions. The datasets with the highest resolutions are reported here in this work.
Randomization	Randomization is not relevant to structural data or ELISAs.
Blinding	The same group of investigators designed and performed the experiments and analyzed the data for all results.

Reporting for specific materials, systems and methods

We require information from authors about some types of materials, experimental systems and methods used in many studies. Here, indicate whether each material, system or method listed is relevant to your study. If you are not sure if a list item applies to your research, read the appropriate section before selecting a response.

Materials & experimental systems

n/a	Involved in the study	
☐	☒	Antibodies
☐	☒	Eukaryotic cell lines
☒	☐	Palaeontology and archaeology
☒	☐	Animals and other organisms
☒	☐	Clinical data
☒	☐	Dual use research of concern

Methods

n/a	Involved in the study	
☒	☐	ChIP-seq
☒	☐	Flow cytometry
☒	☐	MRI-based neuroimaging

Antibodies

Antibodies used

Antibodies used in this study include 17b Fab and JR-52 IgG.

For ELISA data, THE™ His Tag Antibody conjugated to to horse-radish peroxidase (GenScript) was used. Details of usage were described in the Methods section of this manuscript.

Validation

The structural binding mechanisms and binding affinities or HIV-1 Env-targeting antibodies have been described in previous literature:

Dam, K.-M. A., Mutia, P. S. & Bjorkman, P. J. Comparing methods for immobilizing HIV-1 SOSIPs in ELISAs that evaluate antibody binding. Sci. Rep. 12, 11172 (2022).

Yang, Z., Wang, H., Liu, A. Z., Gristick, H. B. & Bjorkman, P. J. Asymmetric opening of HIV-1 Env bound to CD4 and a coreceptor-mimicking antibody. Nat. Struct. Mol. Biol. 26, 1167–1175 (2019).

Ozorowski, G. et al. Open and closed structures reveal allostery and pliability in the HIV-1 envelope spike. Nat. Publ. Group 547, 360–363 (2017).

The binding specificity for THE™ His Tag Antibody conjugated to to horse-radish peroxidase (GenScript) has been validated through a commercial source. The effectiveness of this antibody has been repeatedly tested in ELISA experiments in our lab.

Eukaryotic cell lines

Policy information about cell lines and Sex and Gender in Research

Cell line source(s)

Expi293F cells (Thermo Fisher)

Authentication

Cell lines were not authenticated in the lab.

Mycoplasma contamination

No contamination.

Commonly misidentified lines (See ICLAC register)

No commonly misidentified lines were used.
